# The effect of 1 mg folic acid supplementation on clinical outcomes in female migraine with aura patients

**DOI:** 10.1186/s10194-016-0652-7

**Published:** 2016-06-23

**Authors:** Saras Menon, Bushra Nasir, Nesli Avgan, Sussan Ghassabian, Christopher Oliver, Rodney Lea, Maree Smith, Lyn Griffiths

**Affiliations:** Genomics Research Centre, Institute of Health and Biomedical Innovation, Queensland University of Technology, Kelvin Grove, QLD Australia; Centre for Integrated Preclinical Drug Development Faculty of Medicine and Biomedical Sciences, University of Queensland, Brisbane, QLD Australia; Blackmores Institute, 20 Jubilee Avenue, Warriewood, NSW 2102 Australia

**Keywords:** Migraine, Folate, Folic acid, Vitamin B_6_, Vitamin B_12_

## Abstract

**Background:**

Migraine is a common neurovascular condition that may be linked to hyperhomocysteinemia. We have previously provided evidence that reduction of homocysteine with a vitamin supplementation can reduce the occurrence of migraine in women. The current study examined the occurrence of migraine in response to vitamin supplementation with a lower dose of folic acid.

**Methods:**

This was a 6 month randomised, double blinded placebo controlled trial of daily vitamin supplementation containing 1 mg of folic acid, 25 mg of Vitamin B_6_ and Vitamin B_12,_ on reduction of homocysteine and the occurrence of migraine in 300 female patients diagnosed with migraine with aura.

**Results:**

Vitamin supplementation with 1 mg of folic acid, did not significantly decrease homocysteine levels (*P* = 0.2). The treatment group did not show a significant decrease in the percentage of participants with high migraine disability, severity or frequency at the end of the 6 month intervention (*P* > 0.1).

**Conclusion:**

1 mg of folic acid in combination with vitamin B_6_ and B_12_ is less effective in reducing migraine associated symptoms compared to the previously tested dosage of 2 mg folic acid in combination with 25 mg of vitamin B_6_ and 400 μg of vitamin B_12._

## Background

Migraine is a very common episodic neurological disorder which is typically characterised by attacks of 4 to 72 h of severe headache and associated with autonomic and neurological symptoms [1]. The International Headache Society (IHS) has classified migraine into migraine with aura (MA) and migraine without aura (MO) [2]. The incidence of migraine is much higher in females (70 %) than in males (30 %) and is at its highest during the peak reproductive years (between the ages of 25 and 55 years) [3]. Despite the high prevalence of migraine, its pathophysiology is not completely understood. The activation of the trigeminovascular system which results in vasodilation of pain producing intracranial blood vessels is thought to be responsible for the typical pain migraineurs experience [4]. Family and twin studies have shown that both common and rare migraine subtypes have a significant genetic basis with environmental interactions also playing an important role in disease manifestation [1]. Previous studies have identified neurological, vascular and hormonal pathways to be involved in migraine susceptibility and pathophysiology [5, 6].

The human methylenetetrahydrofolate reductase (MTHFR) gene is involved in the remethylation of homocysteine to methionine. The C677T allele (rs1801133), a common variant of the MTHFR gene has a frequency of approximately 23–41 % in the Caucasian population [7]. The TT genotype of the MTHFR C677T SNP has been shown to be associated with a 50 % reduction in enzyme activity and consequently moderately increased levels of circulating homocysteine [7–10]. This variant and elevated homocysteine levels have been associated with the risk of several neurological conditions including migraine [11, 12]. Homocysteine related endothelial dysfunction might be involved in the initiation and maintenance of a migraine attack. Several studies have shown the TT genotype of the MTHFRC677T variant to significantly increase the risk of MA [8, 9].

Our laboratory has previously investigated the homocysteine-lowering effects of vitamin supplementation in MA patients and the modifying effect of C677T genotype on treatment response. In the Phase 1 trial, the effects of 2 mg folic acid, 25 mg vitamin B_6_ and 400 μg vitamin B_12_ were examined in 52 MA sufferers [13]. The major finding of this study was that vitamin supplementation reduced plasma homocysteine levels and migraine disability significantly in this migraine group. This study also provided some evidence that the effect of vitamin supplementation on reduction of homocysteine levels and migraine disability was influenced by the MTHFRC677T genotype, whereby carriers of the C allele experienced a greater response compared to TT genotypes [13].

In the Phase 2 trial we replicated the treatment options in an independent and larger sample of MA sufferers and examined the genotypic effects of both the MTHFR and MTRR (5-Methyltetrahydrofolate-Homocysteine Methyltransferase Reductase) gene on the reduction of homocysteine and migraine disability in response to vitamin supplementation [14]. The Phase 2 trial provided further evidence that the MTHFR variant influences treatment response and that the MTRR variant may also be acting independently to influence vitamin treatment response in migraineurs. Despite improvements in some participants, the more functionally affected MTHFR T allele and MTRR G carriers seemed to resist homocysteine reduction and did not respond as well in terms of alleviation of migraine symptoms [14]. The purpose of this study was to determine if a lower folic acid dosage (1 mg) that lies within the currently recommended daily intake range was also effective in reducing homocysteine levels and migraine disability. This would enable the development of safe, personalised treatment and prophylactic regimes for migraine in the community.

## Methods

### Patient group

The study recruited female Caucasian adult participants. European descents living in Australia, having emigrating ancestors within the last 160 years from various locations within the British Isles and other parts of Europe were recruited from East Coast of Australia and were interviewed and completed a detailed questionnaire that was administered through the Genomics Research Centre (GRC).

As migraine is more prevalent in females and this prevalence reduces drastically in males only females between the ages of 18 and 60 were recruited. Participants were included if they had suffered migraine for over > 5 years and had a current diagnosis of MA (>90 % of their migraine attacks were associated with aura), and a 1-year history of severe, long lasting attacks (at least 4 attacks lasting more than 48 h), had a family history of migraine. Confirmation of migraine diagnosis was carried out using the IHS criteria. As this hypothesised that the outcome variable, the response to vitamin therapy is influenced by inherited factors, genetic independence of participants was given important attention. Thus it was made sure that only participants that are not related by first degree were included in the study. Participants who were currently taking vitamin supplementation, were pregnant, or had been diagnosed with a clinically recognised co-morbid disease such as vascular disease, depression or epilepsy were excluded from the trial to reduce clinical and pathological heterogeneity. Participants that had taken part in another clinical trial or had received any experimental therapy within the last one month were also excluded from the trial. The patient group was not selected on the basis of pre-existing folic acid, B_12_ or B_6_ deficiency. This study was originally approved by Griffith University Human Research Ethics Committee (HREC), approval number MSC/09/05/HREC.

### Randomisation and blinding

Three hundred female patients meeting the inclusion criteria were randomly assigned into either the placebo or the treatment group. A blocked random allocation sequence was generated using nQuery Advisor (Statistical Solutions, Cork, [Ireland]). Patients and everyone involved in this trial were blinded to randomisation and group allocation. One hundred random female patients were assigned to the placebo group and 200 random female patients were assigned to the vitamin tablet. The numbers in the treatment group were higher to increase the power for genotype comparisons in relation to treatment response.

### Treatment

Patients received either vitamin tablets containing 1 mg of folic acid, 25 mg of vitamin B_6_ and 400 μg of vitamin B_12_ or the placebo tablet. Both the vitamin and placebo tablets were produced by Blackmores (Warriewood, New South Wales, Australia) and were indistinguishable in appearance. Patients were instructed to take one tablet daily for 6 months.

### Baseline and follow-up assessment

Before the treatment all participants were assessed for migraine disability using the Migraine Disability Assessment Score (MIDAS) instrument, which provides a measure of productive days lost to migraine headache in previous 3 months (i.e. migraine disability), as well as headache frequency and pain severity [13, 15]. Patients were asked to complete a daily diary during the trial period to record the details of their migraine symptoms (duration, frequency and severity) and treatment compliance. Patients were also instructed to take their usual migraine treatment for acute attacks. A blood sample was collected for baseline measurement of plasma homocysteine (μmol/l), folate (nmol/l), vitamin B_6_ and B_12_ (pmol/l) concentration. 2 ml of venous blood was collected for Genomic DNA extraction and genotyping purposes.

Patients were contacted after 3 months for headache diary and compliance checking. At the end of the 6 month trial the patients were reassessed at the GRC clinic. They were questioned about their migraine history in the last 6 months since the start of the trial. A second collection of blood samples was done for measurement of homocysteine, folate, B_6_ and B_12_ concentrations.

### Clinical outcome measures

Migraine disability measured by the MIDAS instrument was the primary clinical outcome in this trial. Studies have shown that the MIDAS instrument is a valid and clinically useful instrument for assessing health-related quality of life in migraineurs. Based on the 5-question MIDAS rating, participants were arbitrarily categorised into a ‘low’ disability group if they had a MIDAS rating of 0–10 and into a ‘high’ disability group if they had a MIDAS rating greater than 11 [13, 15, 16]. Secondary outcome variables, which are partly captured within the primary outcome, were migraine frequency and head pain severity. These were measured as number of days with headache (over a 3 month period) and a pain score (based on a scale of 1–10), respectively [13, 15, 16].

### Predictor variables

The primary predictor variable for this study was the treatment groups (Vitamin vs placebo). The secondary predictor variables include plasma homocysteine levels and the C677T polymorphism of the MTHFR gene, grouped by TT and CT and CC genotypes. The C677T polymorphism was genotyped in the patient group in the GRC laboratory using previously published methods [9]. The Plasma homocysteine, folate, B_6_ and B_12_ levels were measured in an accredited pathology laboratory (TetraQ, University of Queensland, Brisbane) [17, 18].

### Statistical analysis

The analysis for the trial was conducted on a modified intention-to-treat (ITT) principle. The modified ITT cohort was composed of all randomised participants who started the trial and consumed study supplements on at least one occasion, excluding those who withdrew from the trial after the randomisation process had taken place but before the commencement of study supplement consumption. At baseline and follow-up time points, unpaired samples *t*-tests were used to test the group means and proportions were compared using the χ^2^ test of independence. The significance threshold was set at α level of 0.05. All analyses were performed using the Statistical Package for Social Sciences (SPSS version 18.0; International Business Machines, Chicago, Illinois, USA).

## Results

**Fig. 1:**
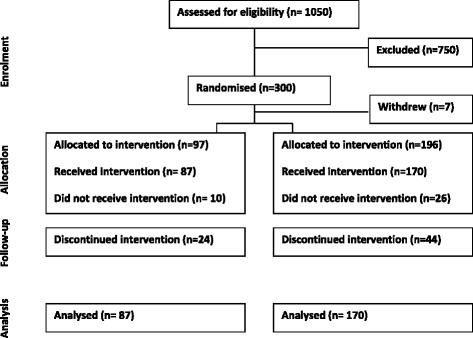
Patient flow chart of the trial

Figure 1 shows the participant flow through the trial. A total of 1050 migraineurs were assessed for eligibility before enrolment into the trial. Of them 750 migraineurs were excluded because of reasons such as nonfulfillment of the inclusion criterial, refusal to participate in a placebo-controlled trial and other reasons. In total, 300 participants were initially enrolled in the trial and were randomly assigned in the ration of 1:2 to either the placebo group or the vitamin- treated group respectively, but seven participants dropped out before the commencement of the trial and 36 participants dropped out at baseline assessment. Two hundred and fifty seven participants received baseline assessment and successfully commenced trial. One hundred and seventy participants were included in the vitamin- treated group and the remaining 87 participants were included in the placebo- treated group. Sixty eight participants were lost to follow-up because of lack of compliance and 189 participants completed the trial (126 Vitamin treated, 63 Placebo treated).

### Baseline analysis

Table 1 shows the baseline clinical characteristics of the participant group. The mean folate concentration was 33.7 nmol/l and 35.6 nmol/l for the placebo and the treatment group respectively, which were above the average folate concentration of 13.7 nmol/l in the general Caucasian population. The mean plasma homocysteine concentration was 9.5 μmol/l and 9.4 μmol/l for the placebo and the treatment group respectively, which were slightly above the average plasma homocysteine level of 8.9 μmol/l in the general Caucasian population. The mean B_6_ and B_12_ levels at baseline for both the placebo and treatment groups were within the normal range for a general Caucasian population. The percentage of participants with high migraine frequency and high migraine severity did not significantly differ between the placebo and the treatment group at baseline, however the percentage of participants with high migraine disability significantly differed between the placebo and the treatment group (*P* =0.02). The placebo group had an increased proportion of participants (91 %) with high migraine disability compared to the treatment group (79 %).

**Table 1:** Clinical characteristics of patient groups at baseline

Analyte	Placebo (*n* = 87)	1 mg (*n* = 170)
Folate	33.7 (11.6)	35.6 (11.9)
B_6_	97.2 (7.3)	132.2 (12.1)
B_12_	266.1 (94.3)	289.3 (104.4)
Hcy	9.5 (2.6)	9.4 (2.8)
High Disability	79 (91 %)	134 (79 %)
High Frequency	56 (64 %)	109 (64 %)
High Severity	43 (49 %)	73(43 %)

### Values are mean (SD) or n (%)

For the blood biochemistries at follow-up (Table 2) - folate, B_6_ and B_12_ levels were significantly higher in the treatment group compared to placebo group (*P* < 0.0001) after 6 months of intervention. Plasma homocysteine levels remained at 9.5 μmol/l for the placebo group while in the treatment group, plasma homocysteine levels decreased from 9.4 to 8.5 μmol/l after the 6 months of intervention. However this decrease was not statistically significant (*P* = 0.2). This result is in contrast to our previous results testing the effect of 2 mg of folic acid, which showed a statistically significant reduction in mean homocysteine (*P* < 0.001) in the treatment group. The baseline Hcy plasma levels of the treatment group was lower in the current trial (9.4 μmol/l) compared to the previous trial, this is most likely due to the higher level of baseline folate levels in the current trial (35.6 nmol/l) compared to the previous trial (31.4 nmol/l), which displays a possible floor effect.

**Table 2:** Summary of blood biochemistry measures at follow-up*

Analyte	Placebo (*n* = 63)	1 mg (*n* = 126)	*P* value*
Folate	34.6 (1.6)	52.6 (0.40)	<0.0001
B6	134.6 (21.5)	490.6 (24.9)	<0.0001
B12	274.1 (11.6)	456.8 (11.6)	<0.0001
Hcy	9.5 (0.32)	8.5 (0.49)	0.2

Values are means (SEM)

Change in folate, B_6_,B_12_ and Hcy levels in the treatment and placebo group after 6 months of intervention. Hcy levels did not significantly decrease in the treatment group compared to the placebo group after 6 months of intervention

*P values were determined by *t*-test

For the migraine clinical outcomes at follow-up (Table 3) the treatment group had a lower percentage of participants with high migraine disability, severity and frequency compared to the placebo group at the end of the 6 month intervention. However, these differences were not statistically significant (*P* > 0.1).

**Table 3:** Summary of migraine outcomes at follow-up*

Analyte	Placebo (*n* = 63)	1 mg (*n* = 126)	*P* value*
(a) High Disability	34 (52 %)	56 (45 %)	0.4
(b) High Frequency	28 (43 %)	44 (36 %)	0.33
(c) High Severity	12 (19 %)	16 (13 %)	0.31

Values are n (%)

High disability (a) is defined as a MIDAS score >11, High Frequency (b) is defined as a score >10 (median) and High Severity (c) is defined as a score >7 (median). All scores were categorised due to the deviation from non-normal distribution and to improve interpretation

*P value were determined by Chi-square tests

When the treatment group was stratified by the MTHFR C677T genotype, an overall decrease in the percentage of high migraine disability, frequency and severity was observed for all three genotype groups after the 6 months of intervention however the decrease was not statistically significant(*P* > 0.10).

## Discussion

Migraine is a chronic and debilitating condition that has significant impact on both the sufferer and the society at large [19]. Although there are several migraine therapies and medications currently available to treat migraine, most of them work with differing efficacy and are often associated with adverse effects [20]. Migraine research thus continues to explore new and/or improved migraine treatments that are both effective and safe for use by all migraineurs [21].

The Genomics Research Centre had previously tested the effects of folate and vitamin B treatment response in migraineurs in two previous studies [13, 14]. The results of these previous studies and have reported the significant decrease in migraine associated symptoms after a 6 month intervention of a vitamin tablet containing 2 mg folic acid, 25 mg of vitamin B_6_ and 400 μg of vitamin B_12 ._ When the effect of the MTHFRC677T and the MTRR A66G genetic variants on migraine treatment was assessed, it was observed that both these variants exert an independent effect on migraine treatment response [14].

The current trial investigated the effect of 1 mg folic acid, 25 mg of vitamin B_6_ and 400 μg of vitamin B_12_ on migraine associated symptoms on adult Caucasian females with MA. The aims of the current study was to investigate the effectiveness of a vitamin supplementation incorporating 1 mg folic acid, 25 mg of vitamin B_6_ and 400 μg of vitamin B_12_ in significantly reducing migraine associated symptoms and to investigate the effect of the MTHFRC677T variant on the reduction of homocysteine and migraine associated symptoms in response to the vitamin supplementation.

The results of this trial did not observe a significant reduction in homocysteine levels in the treatment group compared to the placebo group (*P* = 0.2) and there was also no significant decrease observed in migraine disability, frequency or severity in treatment group compared to the placebo group at the end of the treatment period. This result was in contrast to the previous trials which investigated vitamin supplementation of 2 mg folic acid, 25 mg of vitamin B_6_ and 400 μg of vitamin B_12_ and reported significant decrease in homocysteine levels as well as migraine associated symptoms compared to the placebo group at the end of treatment period. Similarly while the previous trial reported migraine associated variants, in the MTHFR and MTRR genes to have a significant effect on migraine treatment response, the current trial did not observe any significant effect of the MTHFR C677T gene variant on migraine treatment response. This current study has provided evidence that the folic acid dosage in the proposed vitamin supplementation for migraine treatment plays a pertinent part in reducing homocysteine levels and migraine associated symptoms.

Folate, a water-soluble vitamin, includes endogenous food folate and its synthetic form, folic acid [22]. Folate lacks stability in its naturally occurring form in food storage and preparation; however, folic acid is stable and used for supplements and food fortification [23]. There are many critical cellular pathways dependent on folate as a one-carbon source, including DNA, RNA, and protein methylation, as well as DNA synthesis and maintenance [24]. A number of genetic polymorphisms affect critical components of folate pathways and metabolism, and have been associated with an increased risk for several diseases [25–31]. Mandatory folate fortification of wheat flour was implemented in Australia in 2009. The tolerable upper intake level (UL) (1000 μg/day)of folic acid was established as one-fifth of the lowest observed adverse effect level (5000 μg/day) associated with a potential adverse outcome [32]. Even at dosages of 15,000–100,000 μg of folic acid daily, limited evidence was found of direct toxicity from folic acid [33–38]. However recently the possibility that folic acid intake might lead to changes in epigenetic patterns and thus the plausible hypotheses of how folic acid might affect certain diseases has led to concerns about higher folic acid intake and food fortification programs.

The most important concern has surrounded cancer as the field of epigenetics has been studied largely in the context of tumorogenesis where clear DNA methylation pattern changes in tumors have been reported [39–44]. It has been suggested that early exposure to folic acid might prevent some cancers and in contrast, after the development of tumors, higher intake of folic acid might promote growth of existing tumors [45]. However, a very recent meta-analysis of randomised clinical trials (RCTs) of the effects of B vitamins on 37,485 individuals with existing cardiovascular disease showed no increased risk of cancer incidence. Additionally, since the implementation of mandatory folic acid fortification in the United States in 1998, both incidence and mortality of colorectal cancer have continued to decline [46]. With such varying studies results, extensive research is warranted to fully understand the effect of folic acid on disease risk.

Further research is thus needed to understand the use of vitamin supplementation incorporating various doses of folic acid, vitamin B_6_ and B_12_ in regards to not only on the reduction of migraine associated symptoms but also the long term effects of vitamin supplementation on the general well-being of migraineurs.

## Conclusion

The recommend dosage of 1 mg folic acid in combination with vitamins vitamin B_6_ and B_12_ is less effective in reducing migraine associated symptoms compared to the previously tested dosage of 2 mg folic acid in combination with 25 mg of vitamin B_6_ and 400 μg of vitamin B_12._
